# Nanohexagonal iron barium titanate nanoparticles surface-modified NiFe_2_O_4_ composite screen-printed electrode for enzymatic glucose monitoring

**DOI:** 10.1039/d4ra06689h

**Published:** 2024-11-01

**Authors:** Hend S. Magar, Amany M. El Nahrawy, Rabeay Y. A. Hassan, Ali B. Abou Hammad

**Affiliations:** a Applied Organic Chemistry Department, National Research Centre (NRC) Dokki Giza 12622 Egypt hendamer2000@yahoo.com +201121926682; b Solid State Physics Department, Physics Research Division, National Research Centre 33 El Bohouth St., Dokki Giza 12622 Egypt; c Biosensors Research Lab, University of Science and Technology (UST), Zewail City of Science and Technology 6th October City Giza 12578 Egypt

## Abstract

A nanocomposite of iron barium titanate/NiFe_2_O_4_ (FBT/NF) was synthesized using sol–gel techniques to form organized hexagonal structures. The effects of NiFe_2_O_4_ nanostructures on FBT's phase purity, morphology, and dielectric properties were systematically explored and intensively discussed. TEM imaging confirmed the hexagonal structure, and electrical measurements revealed that para-electric NF influenced the conductivity and impedance of ferroelectric FBT, with a shift in Curie temperature to lower values. The FBT/NF nanocomposite was optimized for use in glucose amperometric biosensors, offering fast and direct electron transfer from glucose oxidase that was chemically immobilized on disposable sensor chips. Thus, the biosensor exhibited high sensitivity (757.14 μA mM^−1^ cm^−2^), a fast response time of 50 seconds, and a wide linear range of 0.0027–1.9 mM with a low detection limit of 0.5 μM. Accordingly, the biosensor was exploited for blood glucose detection, showing high precision compared to reference methods. These findings highlighted the potential of the FBT/NF nanocomposite for use in developing biosensor portable devices.

## Abstract 2

Nanocomposites of iron barium titanate/NiFe_2_O_4_ (FBT/NF) were fabricated and used as nanostructure-based electrochemical biosensors for glucose detection in human blood samples with high precision compared to reference methods.

## Graphical abstract

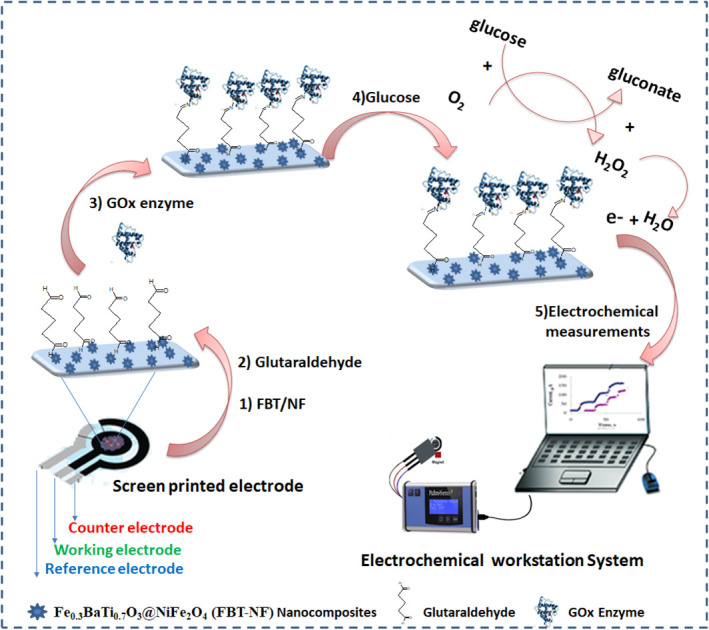

## 1 Introduction

Over the past few decades, there has been significant research focus on multiferroic-versatile materials that concurrently display ferroelectricity and magnetism within a single structure.^1–4^ This intense study stems from their promising potential applications in versatile promising fields including data storage, water treatment, energy storage, electromagnetic wave absorption, phase shifting, magnetoelectric (ME) actuators, transducers, and biosensors.^5–7^

Within the field of multiferroics, a specific category of nanocomposites known as perovskite/magnetic metal nanocomposites is emerging as a promising class of nanomaterials to exhibit either a core/shell or random structure, offering a unique avenue to capitalize on both dielectric and magnetic characteristics.^8–11^ These composites exhibit either a core/shell or random structure, providing a unique opportunity to leverage both dielectric and magnetic properties. This potential is realized through careful selection of the specific types of perovskite and magnetic materials. The alteration of BaTiO_3_ materials results in an asymmetric displacement of ions or dipoles within the crystal lattice, leading to a modification in the overall electrical polarization and the generation of piezoelectricity.^3,4^

Until now, diverse magnetic nanoparticles derived from transition metals (such as Co, Fe, Ni) have been combined with dielectric materials like BaTiO_3_, TiO_2_, ZnTiO_3_, ferrite, graphite, and polymers, resulting in successfully prepared nanocomposites with remarkable properties.^12–14^ These findings highlight that the performance of perovskite/magnetic metal nanocomposites is largely determined by their complex microstructure, as well as their permeability, permittivity, and chemical stability.

On the other hand, electron transfer (ET) in biological systems is a very important phenomenon for the areas of biophysical, biochemical and biomedical sciences. In particular, ET is a major regulating factor for bio-electrochemical systems (BESs) including electrochemical biosensors.^15^ Basically, a high-performance biosensor with high efficiency of ET is dependent on the platform's material that is used for immobilization of biomolecules.^16,17^ For instance, in the case of enzyme-based biosensors, denaturation and loss of enzyme bioactivity is resulting from the use of unsuitable platforms for the adsorption of enzymes.^18^ Thus, recent advances for immobilization strategies were employed to develop enzymatic biosensors.^19^ In this regard, the use of nanomaterials provided very efficient alternatives for constructing sensitive and selective biosensors due to the stable, active and well-oriented immobilized biomolecules. Nanostructured materials were categorized as inorganic,^20^ organic^21,22^ or hybrid (organic–inorganic or metal–organic) nanostructures.^23^

The fabricated nanostructures-based electrochemical biosensors were designed for the detection of infectious diseases or for health care diagnosis.^24^ Since the type and structure of nanomaterials have high impact on the performance of BESs, modification of the electrode surface by using nanoparticles (NPs), nanorods, nanotubes or other nanostructures was conducted.^25^ Perovskite/magnetic metal nanocomposites have been exploited for efficient and sensitive biosensors fabrication.^26–28^

On the other hand, better electro-catalytic properties of the perovskite/magnetic metal nanocomposites and the ease of fabrication of their nanostructures make them extremely attractive materials for sensitive biosensing devices. Therefore, the immobilization of target biomolecules such as, cholesterol oxidase, glucose oxidase, urease, cytochrome C, tyrosinase or horseradish peroxidase (HRP) on nanostructured metal oxides was successfully obtained. Piezoelectric BaTiO_3_ materials belong to the category of biomaterials that produce charges in response to slight deformations caused by various modifiers.^29,30^

Herein, we exploited the benefits of piezoelectric material (Fe_0.3_BaTi_0.7_O_3_) and nanomagnetic NiFe_2_O_4_ within a chemical manufacturing process. The objective is to produce glucose biosensors with functionality tailored for accurate glucose monitoring directly in blood samples.

In the current study, Fe_0.3_BaTi_0.7_O_3_@NiFe_2_O_4_ nanohexagnoal composites were developed, characterized and utilized for the direct biosensing of glucose. The nanohexagnoal composites with highly electrocatalytic behavior was implemented to support the interaction between the electrode surface and the active site of the immobilized sensing molecules, *e.g.* glucose oxidase (GOx). Thus, the main goal of this study is the construction of a novel biosensor platform for efficient enzymatic glucose biosensing using the synthesized Fe_0.3_BaTi_0.7_O_3_@NiFe_2_O_4_ nanohexagnoal composites on the screen-printed electrodes. Besides, a synergetic electrocatalytic performance of the developed nanocomposite, fast electron communication between the enzyme's active sites and the screen-printed electrode is expected from the new sensing approach.

## 2 Experimental

### 2.1. Materials

All chemicals were of analytical reagent grade and used as received. Nickel and ferric nitrates (Ni(NO_3_)_2_·6H_2_O, and Fe(NO_3_)_3_·9H_2_O), barium acetate (Ba(CH_3_COO)_2_, 99%), titanium(iv) isopropoxide (Ti[OCH(CH_3_)_2_)], iron nitrate, citric acid, and nitric acid were used as a raw materials were obtained from Sigma-Aldrich. Glucose oxidase (GOx, CAS RN:9001-37-0 number: G0050 from *Aspergillus niger*, 40 U mg^−1^), β-d(+)-glucose, ascorbic acid, dopamine, uric acid and paracetamol were obtained from Sigma-Aldrich (St. Louis, MO, USA). Glutaraldehyde (GA) was obtained from Fluka (Buchs, Switzerland).

Potassium chloride, ferricyanide [Fe(CN)_6_]^3−^, and ferrocyanide [Fe(CN)_6_]^4−^ were purchased from Pio CHEM. Phosphate buffer saline (PBS, tablets with the pH = 7.4) were obtained from MPBio. Commercial screen-printed carbon electrodes with dimensions: 50 × 13 mm (*h* × *w*) were purchased from Zensors Company, where printed electrodes which consist of three parts including carbon as working and counter electrodes and silver as reference electrode (The diameter of working electrode is 3.0 mm of graphitic carbon). All other chemicals and solvents were of analytical grade and were used without further purification.

### 2.2. Apparatus and instrumentation

X-ray diffraction (XRD) with (XPERT, CuKα; *λ* = 0.15405 nm) was achieved to identify the crystalline phases existing in the fabricated nanocomposites.

Transmission electron microscopy (TEM) and high-resolution transmission electron microscopy (HRTEM) were conducted using (FEI TECNAI G2 F20) microscope operating at (200 kV).

Hioki LCR Meter IM3536 was employed to measure the electrical properties of the composite samples in the frequency range from 4 Hz to 8 MHz. Samples were compacted into disks with thickness *d* and 6 mm radius. A parallel electrodes capacitor technique was used to measure the electrical properties of the samples.

The electric conductivity is given by
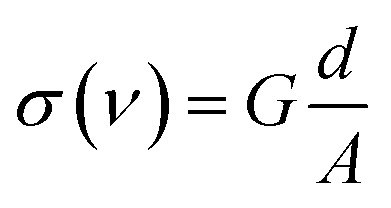
where *A* is the electrode area, 
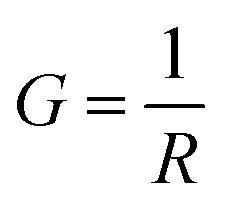
 , and *R* is the resistance in Ω. The electrical impedance of the samples was obtained from the following relationZ* = *Z*′ + *jZ*′′

Therefore, the components of the electrical impedance are given by the following equations.^18,31^
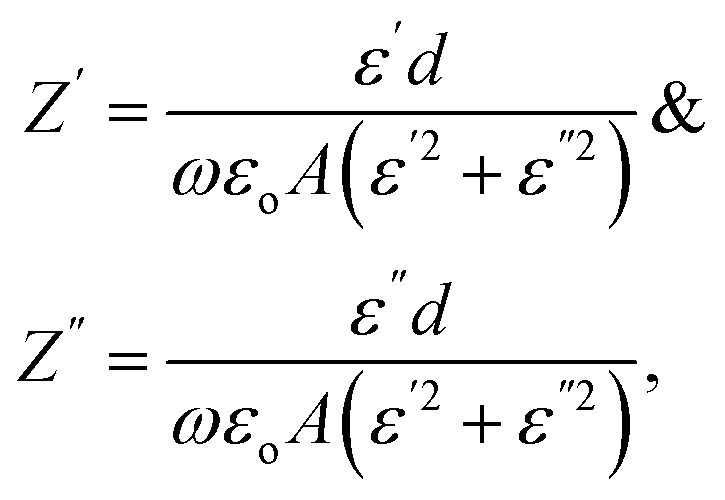
where *ω* = 2π*ν* and 
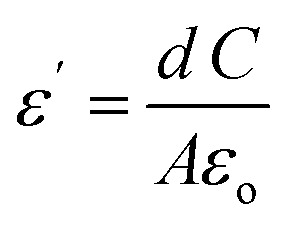
 where *C* is the recorded capacitance.

### 2.3. Methods

#### 2.3.1. Fabrication of nano hexagonal Fe_0.3_BaTi_0.7_O_3_ coated with NiFe_2_O_4_ composites

Nickel ferrite (NiFe_2_O_4_, NF) coated iron barium titanate (FBT) was fabricated through a modified sol–gel process.^32^ This technique facilitates the creation of a multiferroic magnetoelectric material, enabling controlled coexistence of magnetic and electric phases within a single structure.

Nickel and ferric nitrates (Ni(NO_3_)_2_·6H_2_O, and Fe(NO_3_)_3_·9H_2_O) were dissolved individually in deionized water in a 1 : 2 molar ratio in the presence of citric acid. The solutions were then combined and subjected to vigorous stirring for 2 hours at room temperature to achieve a uniformly clear and homogeneous solution before used as a coated layer.

The preparation of hexagonal iron barium titanate nanoparticles involved the sol–gel process, employing specified quantities of barium acetate, titanium(iv) iso-propoxide, and iron nitrate as sources for Ba, Ti, and Fe, respectively. Acetic acid (HAc) and acetylacetone (AcAc, C_5_H_8_O_2_) served as appropriate solvents. Titanium(iv) isopropoxide was dissolved in acetylacetone, while barium acetate and iron nitrate were dissolved in acetic acid/water. The resulting solutions stirred for 1 h, and dried at 250 °C. Subsequent thermal treatment at 500 °C for 2 hours yielded shaped iron barium titanate powder.

For FBT coated with *x*NiFe_2_O_4_ (where *x* = 0, 0.1, 0.3, and 0.5), the resulting FBT nanopowder, post-calcination at 500 °C, was introduced into a NF solution based on the desired weight percentage. The resulting solution underwent stirring for 1.30 min at 70 °C. Upon drying at 250 °C, the solution transformed into a viscous brown gel before transitioning into a xerogel form. Finally, the obtained xerogels underwent calcination at 600 °C. The nanocomposite samples were designated with symbols BN, 1BN, 3BN, and 5BN.

#### 2.3.2. Preparation of enzymatic-based biosensors chips

**Scheme 1:**
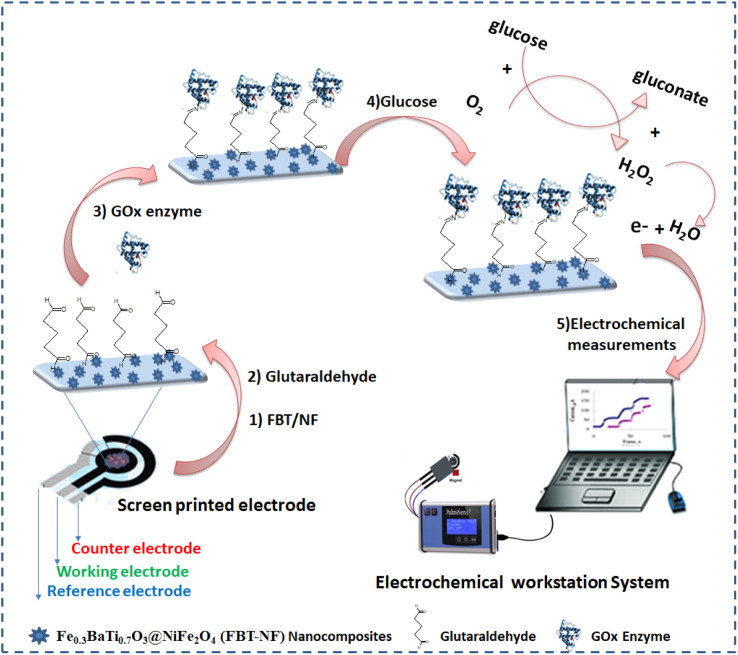
A simple demonstration presented the steps of glucose biosensor preparations showing the chemical immobilization of the active enzyme (GOx) though the crosslinking with the glutaraldehyde molecules. Oxidation of glucose to gluconic acid and generation of peroxide as an electrochemically active bi-product to be oxidized at the active nanomaterial substrate.

The first layer on the SPE was included the formation of a thin layer of the electroactive nanomaterials (FBT/NF@SPE), whereas 5 μl of a dispersed FBT/NF (25 mg ml^−1^) solution was drop casted onto the working part of SPE surface and left to dry at room temperature. Consequently, 5 μl of glutaraldehyde (GA, 2.5%) solution was added to the modified surface of FBT/NF@SPE and then immediately 5 μl of GOx solution (500 μg ml^−1^) was added on GA layer and left to dry at room temperature (as shown in Scheme 1).

The fabricated biosensor (GOx@GA/FBT/NF@SPE) was washed carefully by PBS buffer solution (pH = 7) to remove any unattached enzymes.

In order to optimize the GOx concentration, the enzymatic solutions were prepared by dissolving different amounts of GOx (100 to 700 μg ml^−1^) in phosphate buffer saline(pH = 7.4). The modified electrodes were stored at 4 °C overnight for further experimental work.

#### 2.3.3. Electrochemical characterization of the modified SPE

For electrochemical characterization (CV and EIS) measurements were carried out in a solution containing 0.1 M potassium chloride (KCl) and 5 mM of potassium ferro/ferri cyanide [Fe (CN)_6_]^3−/4−^ as a redox probe. CV studies were carried out with scan rate of 50 mV s^−1^ and potential range from −0.4 to +0.8 V.

The EIS data (which used for understanding the surface structure) were carried out with a range of frequency from 0.1 to 100 000 Hz at open circuit potential under 5 mV potential amplitude and a certain equivalent circuit was selected for fitting the Nyquist impedance plot. Screen printed electrodes and Palmsens4 instrument were used for all electrochemical data measurements.

#### 2.3.4. Biosensor's preparation using different ratios of FBT/NF nanocomposite

Different ratios of the FBT/NF (FBT; FBT:1NF; FBT:3NF; or FBT:5NF) were prepared and used for the SPE modification as the first sensing layer. Whereas, a 5 μl of each of these dispersions was casted onto the printed surfaces before adding the cross-linker layer (5 μl of GA (2.5%)), followed by adding the top sensing layer (5 μl of the GOx enzyme (500 μg ml^−1^)). The modified biosensors were activated by 5 cycles scans using phosphate buffer (pH = 7.4) from −0.5 to +1.0 V then different concentrations of glucose (2.77, 5.55, 8.32, 11.1, 13.8, 16.6, and 19.4 mM) were injected into the electrochemical cell containing a PBS buffer solution (pH = 7.4).

#### 2.3.5. Chronoamperometric analysis

For glucose chronoamperometric measurements, the optimized conditions of GOx@GA@FBT/5NF@SPE biosensor were investigated (such as accumulation potential, enzyme concentrations and pH values of PBS buffer), interferences study and human blood samples analysis.

### 2.4. The application for glucose detection in blood samples

A local clinical lab (El Ahram lab) in Egypt was responsible for collecting and providing the blood samples (Ethical No. 114110923).

A freshly prepared GOx@GA@FBT/5NF@SPE biosensor was applied for glucose analysis in ten blood serum samples of patients without any pretreatments for the samples. A certain volume of the samples (300 μl) was added into the electrochemical cell containing 3 ml of the supporting electrolyte. The glucose concertation values which obtained using the proposed biosensor and chronoamperometric analysis were compared and validated with the values which reported by the clinic laboratory (El Ahram lab).

## 3 Results and discussion

### 3.1. Phase purity of FBT/NF nanostructures

**Fig. 1:**
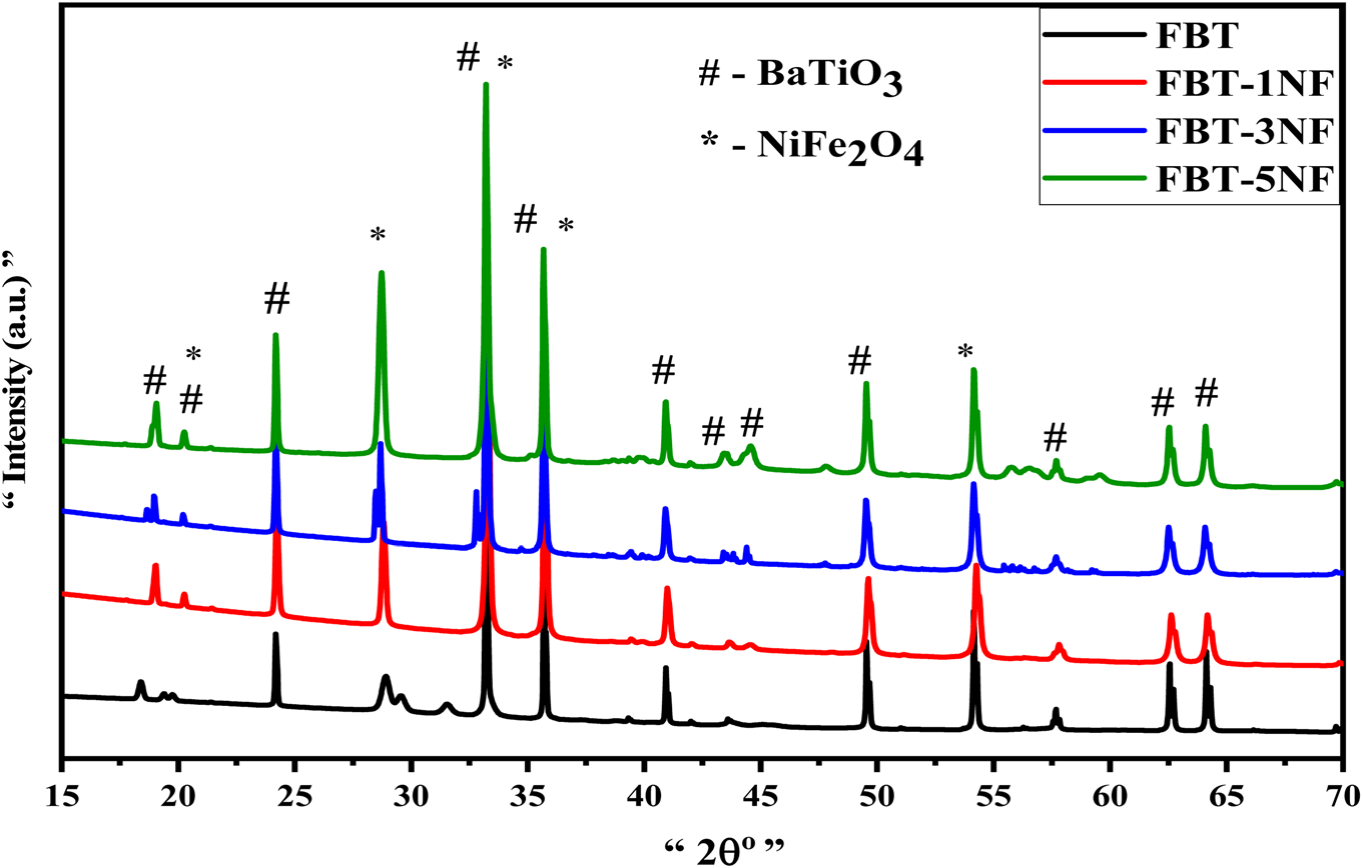
XRD patterns of nano hexagonal Fe_0.3_BaTi_0.7_O_3_ coated by (1, 3, 5) NF composites.

To highlight the distinctive peaks associated with the nanoceramics under consideration, the X-ray diffraction patterns depicted in Fig. 1 show the FBT as a core coated with NF-ferrite nanopowder, focusing on the nanoparticle layer of the FBT core. As shown in Fig. 1, the XRD of pure FBT nanoparticles appeared a crystalline hexagonal BaTiO_3_ phase with the peaks at 19.06°, 20.25°, 24.17°, 33.3°, 35.68°, 40.8°, 43.4°, 44°, 57.7° and 62.5° which are associated with “101”, “102”, “004”, “112”, “201”, “114”, “106”, “108”, and “118” corresponds to the plane of the hexagonal phase (JCPDS: 34-0129) FBT. Fig. 1 (1–5 NF) for FBT/(1–5) NF samples reveal the existence of NiFe_2_O_4_ coated layer, we found an increase in the peaks at 2*θ* = 19.06°, 20.25°, 28.7°, 33.3°, 35.68°, 44°, 57.7° and 62.5° which indexed to the cubic structure of NiFe_2_O_4_ (PDF 03-0875).^33^ Evidently, the presence of hexagonal FBT and cubic NF phases in the growth signifies the reaction of the lower calcination core FBT nanoparticles with the coated NiFe_2_O_4_, resulting in good arrangement within the network structure of Fe_0.3_BaTi_0.7_O_3_@NiFe_2_O_4_ composites. The XRD patterns indicate the presence of diffraction peaks corresponding to both phases, suggesting that the FBT@NF core and the coated layer in the nanocomposites maintain distinct identities in their crystalline structures. This confirms the successful fabrication of di-phase nano hexagonal composite ceramics with minimal chemical reaction occurring at the interfaces between the perovskite and ferrite phases. Furthermore, the average crystallite size (approximately 20–27 nm) of the FBT@NF and pure FBT was calculated using the Scherrer formula.

### 3.2. Morphology of nano hexagonal FBT/NF composites

**Fig. 2:**
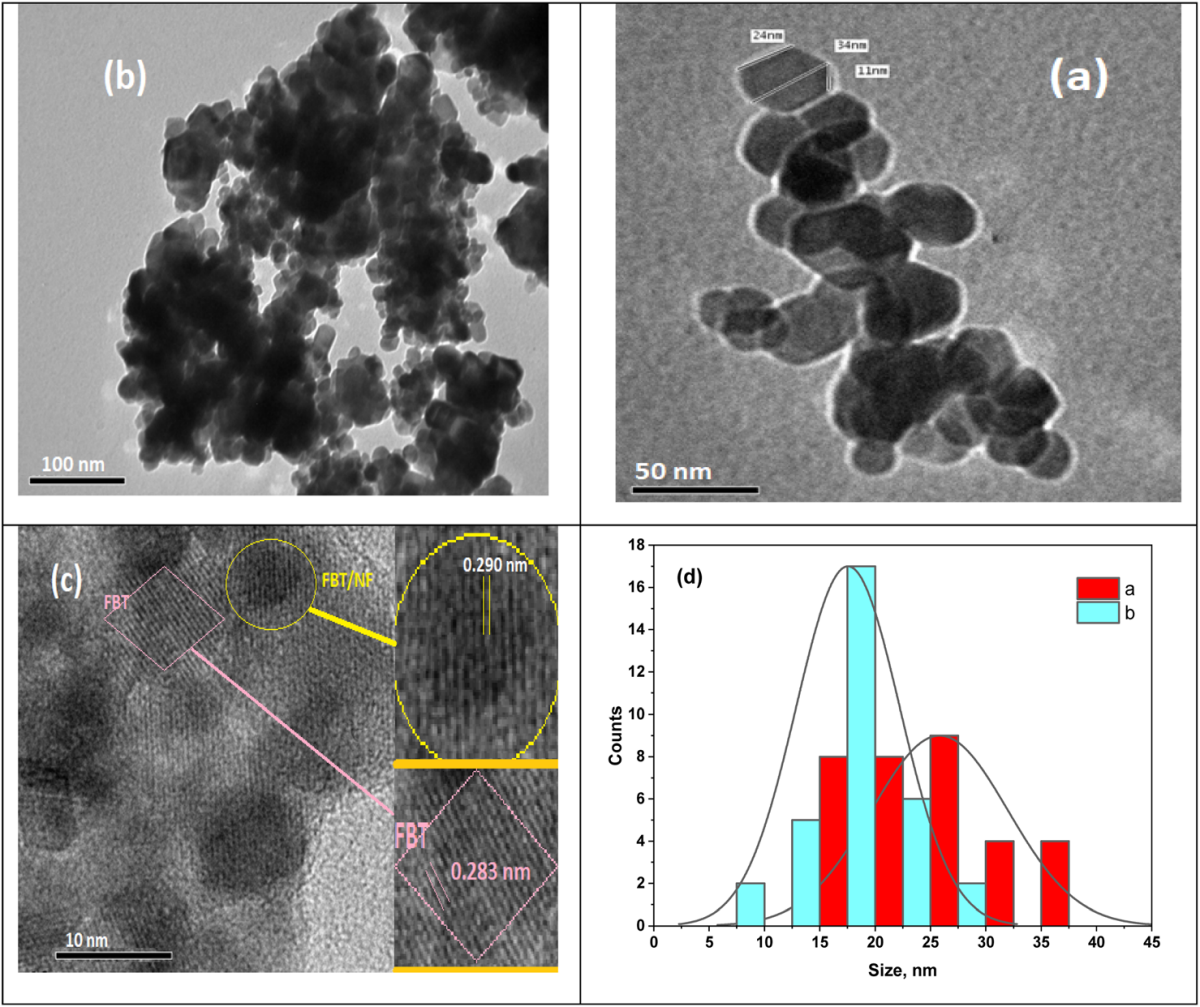
TEM images of (a) FBT, (b and c) 5 NF and (d) size distribution of nanocomposite.

Further insight into the FBT nanoparticles and their surface modification with NF was obtained through transmission electron microscopy (TEM) images presented in Fig. 2. Fig. 2a and b display TEM images of the hexagonal FBT and FBT/5NF samples, while Fig. 2c offers a high-resolution transmission electron microscopy (HRTEM) image of the FBT/5NF sample. The TEM image confirms the well-structured hexagonal arrangement and uniformity of FBT/NF with the existence of highly ordered hexagonal lattice channels, showing a diameter ranging from approximately 20 to 47 nm.

As depicted in Fig. 2c, distinct inter-planers are evident in the image, signaling the existence of a nanostructure in FBT/NF characterized by long-range order.

The HRTEM image of the FBT/5NF sample reveals two distinct interplanar spacings, one measuring 0.283 nm for FBT nanoparticles and the other at 0.290 nm, indicating the growth of FBT/NF. The FBT and FBT/5NF samples display noticeable clustering of hexagonal nanoparticles, potentially matching XRD intensity.

Some agglomerated nanoparticles and dark spots appeared in TEM images resulting from FBT shielding by NF, interactions between magnetic NF nanoparticles, and the higher surface energy of FBT. The formation of clustering behavior suggests that the FBT coating supports the uniformly distributed NF nanoparticles over the surface of the FBT. Also, the agglomerations (Fig. 2c) may arise from the attractive forces resulting from the acquired magnetic properties due to coating with magnetic nanoparticles (NF), leading to their proximity or fusion.^34,35^ The average particle size of the nano hexagons is 18 nm for FBT and 20 nm for FBT/5NF, as illustrated in Fig. 2d aligning well with the observations from the TEM image.

### 3.3. Electrical properties

#### 3.3.1. AC conductivity

**Fig. 3:**
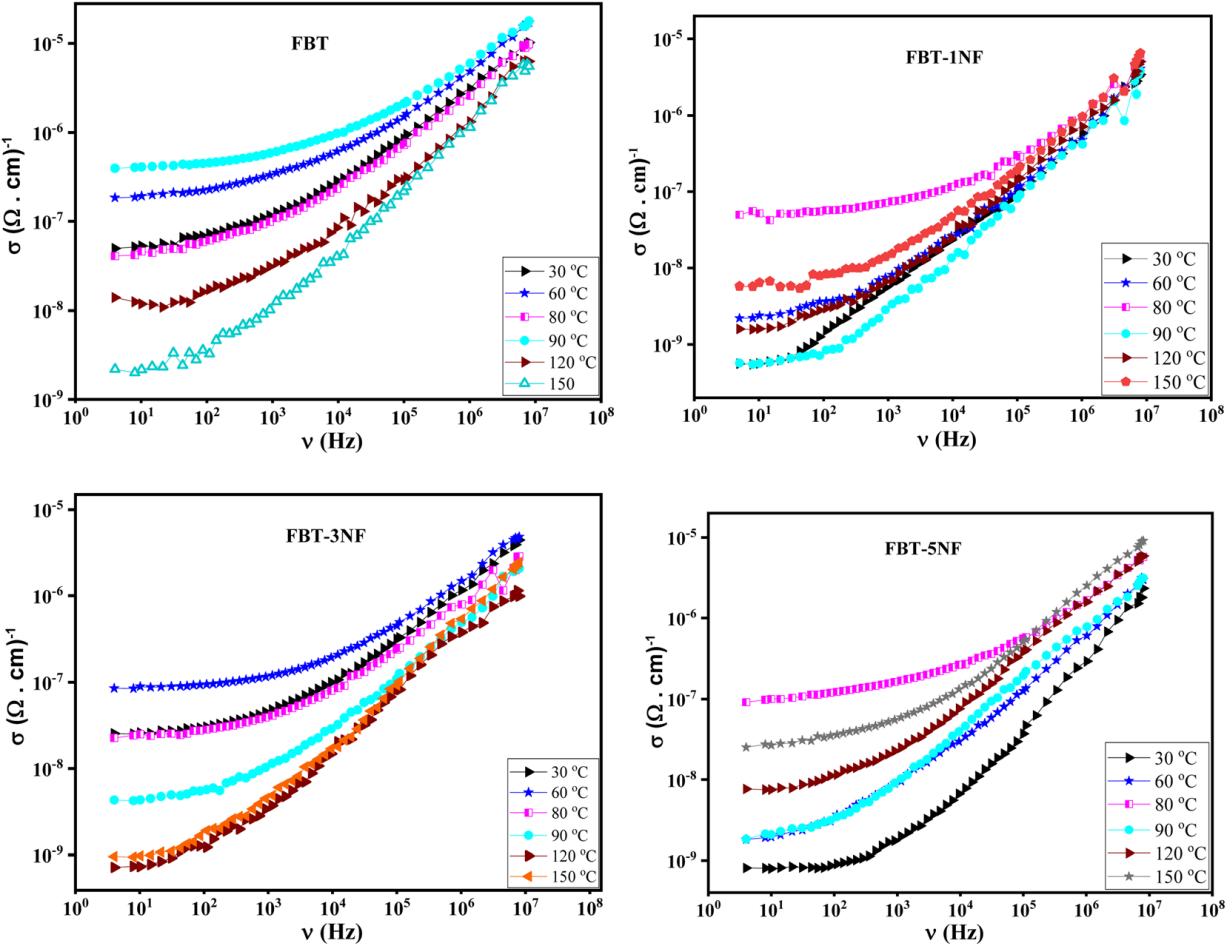
The AC conductivity (*σ*(*ν*)) *versus* frequency at different temperature of iron barium titanate (FBT) and iron barium titanate shielded with nickel ferrite (FBT/*x*NF).

In Fig. 3, the plot illustrates the AC conductivity (*σ*(*ν*)) as a function of frequency at different temperatures for iron barium titanate (FBT) and iron barium titanate shielded with nickel ferrite (FBT/*x*NF). The figure reveals that the AC conductivity in composite samples comprises two distinct components. The first component is observed in the low-frequency region, the conductivity has a steady state with increasing frequency and arises from the direct motion of charge carriers between the conducting electrodes. It represents long-range hopping of charge carriers and is independent of frequency, the DC conductivity contribution (*σ*_DC_).^22–24^ At this range of frequency, the materials behave as a resistor. The second component is observed in the high-frequency region, where the conductivity increases with frequency, it can be attributed to the localized motion of charge carriers, and it represents the conductivity that is released from the dielectric polarization.^25^ At this range of frequency, the material behaves as a capacitor, and the AC conductivity follows Jonscher's power law.^36,37^*σ*_AC_ = *σ*_DC_ + *Aω*^*s*^*A* is the pre-exponential factor and *s* is the power law exponent, 0 < *s* < 1. *A* and *s* depend on the applied temperature. The Jonscher equation was employed to fit the experimental conductivity data, and the resulting data have been presented in Table 1. Examination of Table 1 reveals that the “*s*” values fall within the range of 0.5 to 0.9, providing clear evidence that the movement of these charges is influenced not only by conduction but also by the significant degree of polarization.^27^ Additionally, the declining trend in the “*s*” values suggests that the effective conduction process in the sample corresponds to the Correlated Barrier Hopping (CBH) model.^25^

**Table 1:** The power law exponent at different temperature of the composite samples

Temperature	FBT (*T*_c_ ≈ 90 °C)	FBT-1NF (*T*_c_ ≈ 75 °C)	FBT-3NF (*T*_c_ ≈ 50 °C)	FBT-5NF (*T*_c_ ≈ 75 °C)
	*s*	*s*	*s*	*s*
30	0.76919	0.74	0.6489	0.8578
40	0.61525	0.72429	0.58477	0.7559
50	0.58289	0.7209	0.59787	0.67227
60	0.61085	0.752	0.58419	0.66026
70	0.6014	0.67354	0.59273	0.61619
80	0.60217	0.64476	0.5653	0.6097
90	0.52224	0.7024	0.67184	0.7022
100	0.57845	0.69508	0.6684	0.7163
110	0.62166	0.64528	0.7682	0.7025

The prepared composites belong to the ferroelectric materials. Therefore, they have a unique trend with temperature. As presented in Fig. 3, the conductivity of the prepared composites increases with increasing temperature up to a certain temperature (Curie temperature, *T*_c_) and then decreases with further increase in temperature. This behavior is attributed to increasing the total charge carriers (*e.g.*, electrons or holes) that become thermally excited with increasing temperature below the Curie temperature. Above the Curie temperature, the composite samples lose their spontaneous polarization and transfer to a paraelectric material. Therefore, the conductivity of the composite samples decreases with increasing temperature above Curie temperature. Consequently, the composite samples behave like semiconductors below the Curie temperature and behave like typical dielectric materials above the Curie temperature.^28^

The Curie temperature of the prepared composite is listed in Table 1. It was found that the Curie temperature of the nano-composite decreases with increasing the concentration of the paraelectric phase (NF) which is expected behavior due to the decreasing of the ferroelectric phase and hence decreasing the total number of spontaneous polarizations. The presence of the paraelectric phase hinders the movement and the interaction between the spontaneous dipole moment, which leads to a decrease in the electrical conductivity compared to the conductivity of FBT as observed in Fig. 3.

#### 3.3.2. Bulk impedance

**Fig. 4:**
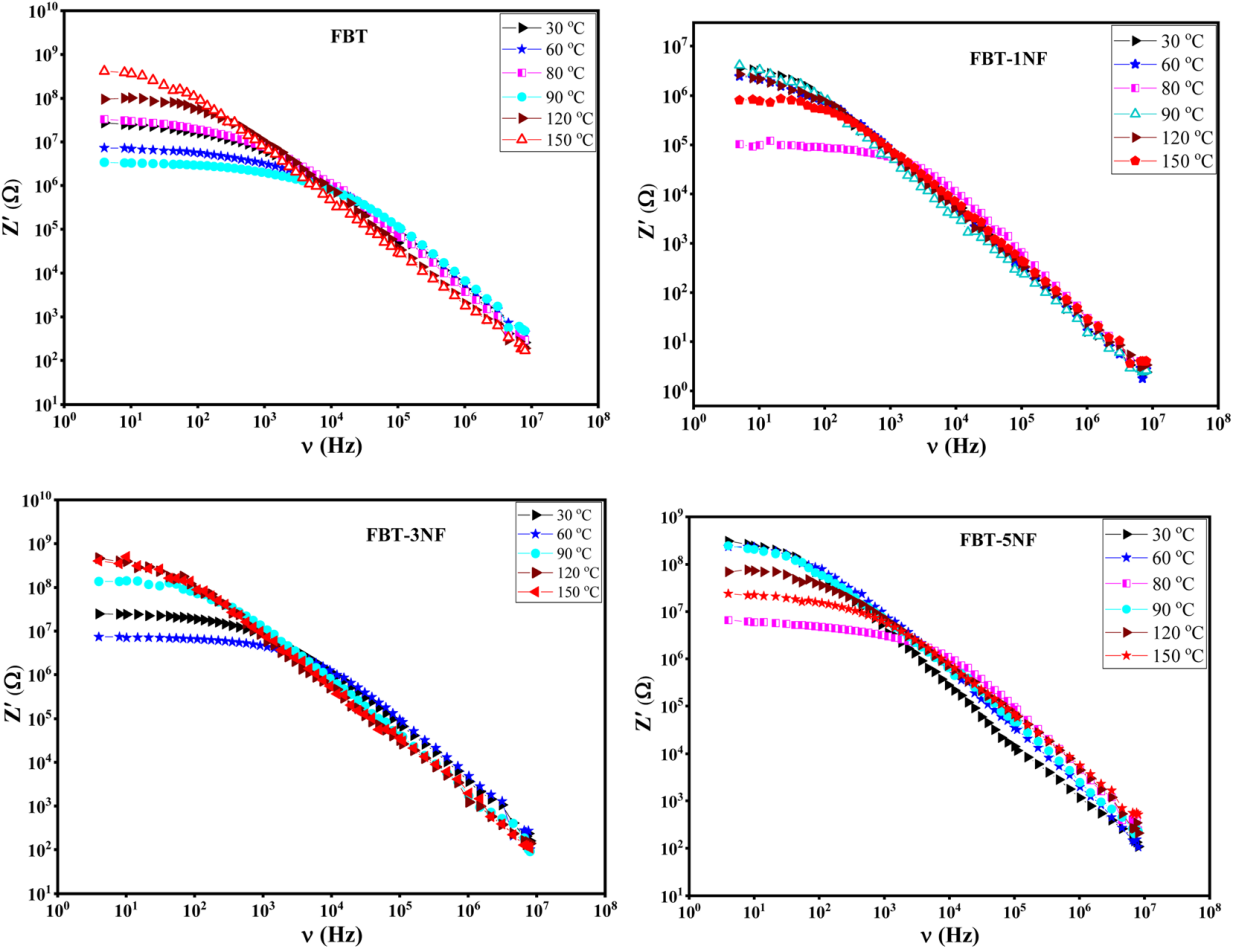
The real impedance (*Z*′(*ν*)) *versus* frequency at different temperature of iron barium titanate (FBT) and iron barium titanate shielded with nickel ferrite (FBT/*x*NF).

The composite nanoceramic materials are suggested to be composed of a paralleling combination of resistor and capacitor. Fig. 4 presented the real impedance (*Z*′(*ν*)) of the composite samples. It shows that the impedance decreases slowly with increasing frequency up to a certain frequency then decrease rapidly after that frequency.^29^ Below this frequency the impedance is the dc resistance effect that released from the resistor while after this frequency the impedance is ac resistance which released from the capacitor. According to Fig. 4 the frequency at which the impedance transforms from the dc to ac shifts to higher frequency with increasing temperature up to the Curie temperature and also the impedance decreases, that confirm the samples are thermally activated and behave like semiconductor. After the Curie temperature, the impedance increases with increasing temperature which suggests samples lose a lot of charge carriers due to the absence of the spontaneous polarization in the paraelectric phase. However, at high frequencies, the real impedance for all temperatures is merged, which can be attributed to the liberation of the charge carriers and reduction of the barrier properties of the composite samples.^25,30^

**Fig. 5:**
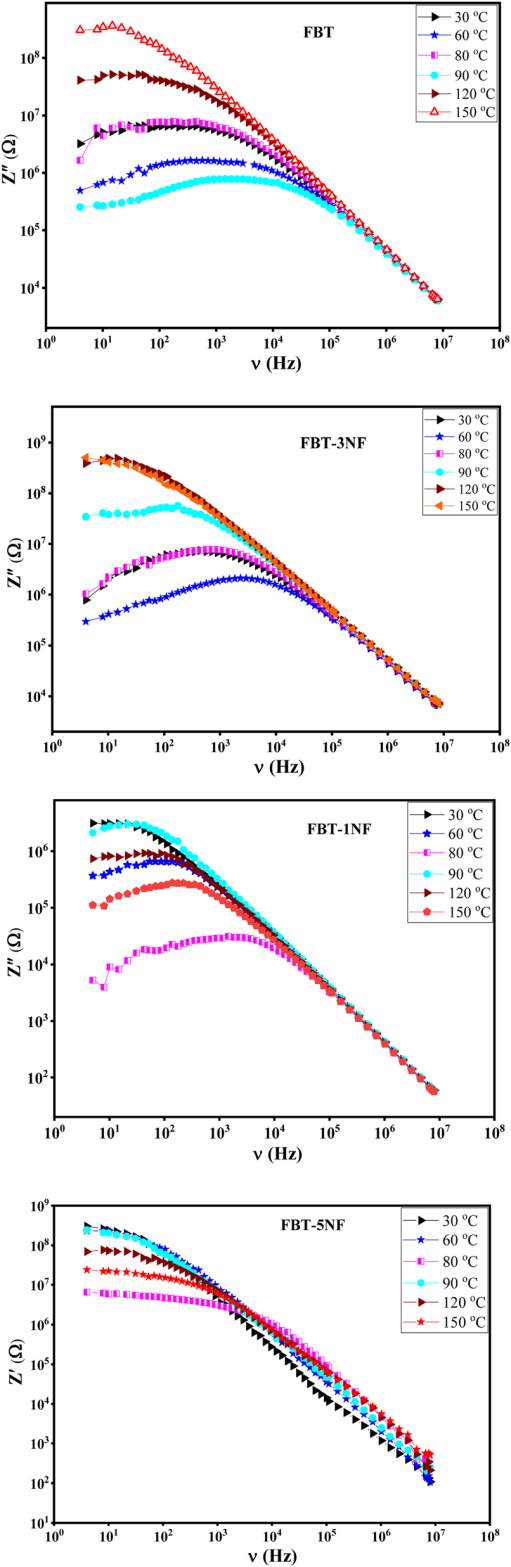
The imaginary impedance (*Z*′′(*ν*)) *versus* frequency at different temperature of iron barium titanate (FBT) and iron barium titanate shielded with nickel ferrite (FBT/*x*NF).

Fig. 5 introduces the imaginary impedance (*Z*′′(*ν*)) of the composite samples at different temperatures. It shows the same behavior as the real impedance with appearance of broad asymmetric peaks, that refer to the non-Debye relaxation in the samples. The behavior of the imaginary impedance with temperature confirms the presence of the ferroelectric to paraelectric phase transition.^38,39^

**Fig. 6:**
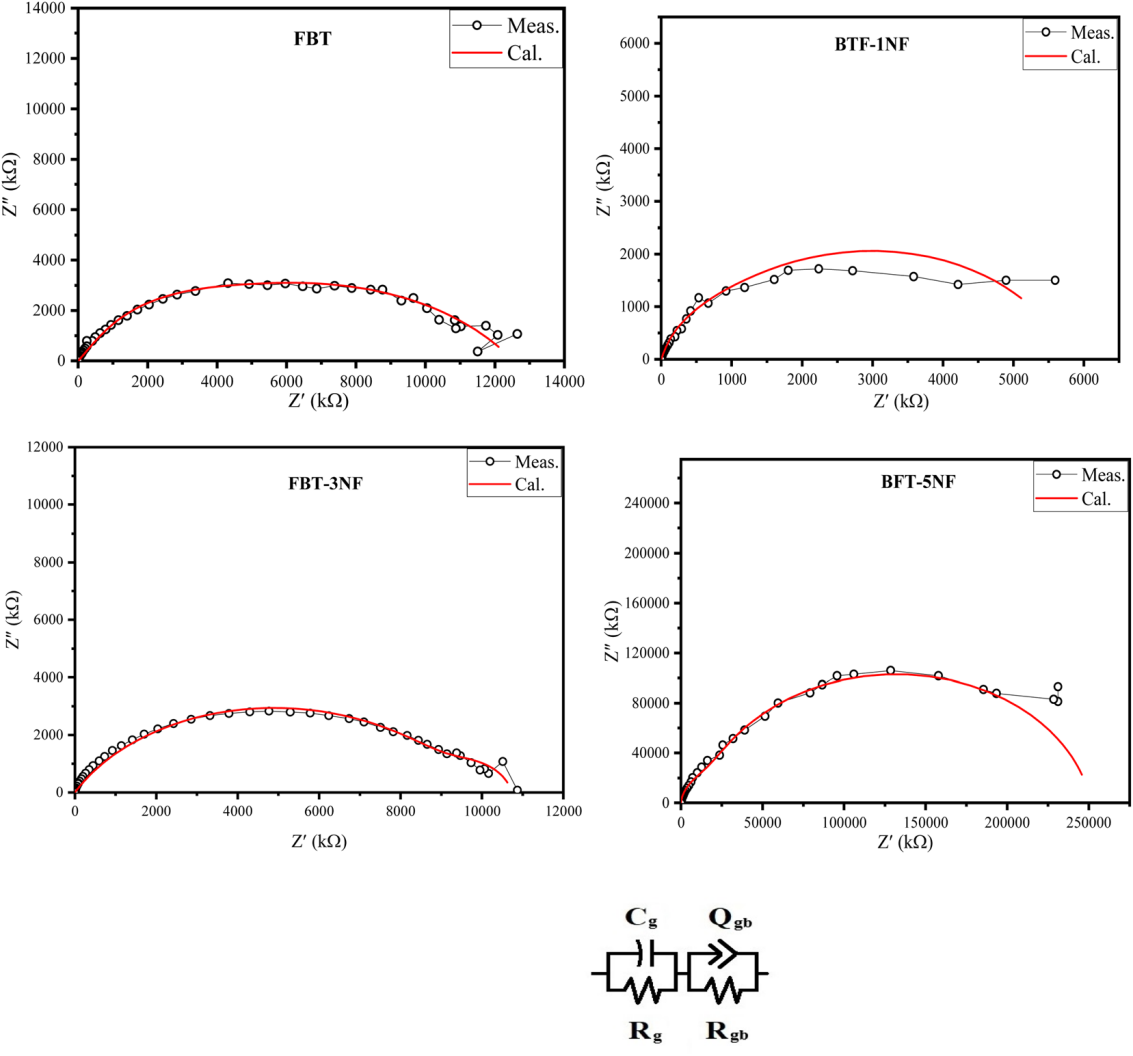
The Nyquist plots at 40 °C and the equivalent circuit of iron barium titanate (FBT) and iron barium titanate shielded with nickel ferrite (FBT/*x*NF).

Fig. 6 represents the Nyquist plots and the equivalent circuit. The Nyquist plots show a depressed semicircle with its center below the real impedance axes, which confirms the non-Debye type of relaxation. Also, the semicircles are unsymmetric. The experimental data were fitted, and the estimated equivalent circuit is plotted in Fig. 6. The equivalent circuit shows the presence of two parallel circuits connected in series; each one is represented by a semicircle, which declares the unsymmetric of the total equivalent circuit due to the overlapping between the two semicircles. Polycrystalline materials are composed of conducting grains and insulator grain boundaries. The effect of grains is predominant at the high frequency, while the impact of grain boundaries is predominant at low frequency under the effect of an AC field. The estimated data are presented in Table 2. *C*_g_ and *R*_g_ represent the effect of the grains, and *R*_gb_ and *Q*_gb_ (p & n) represent the grain boundary effect. The values of the quantum phase element component (p &n) in Table 2 indicate a capacitive effect is dominant in the low frequency.

**Table 2:** The estimated values of the equivalent circuit component

	*C*_g_	*R*_g_ (Ω)	*R*_gb_(Ω)	*Q*_gb_	*n*_gb_
FBT	3.8 × 10^−11^	1.64 × 10^6^	1.1 × 10^7^	8.5 × 10^−10^	0.6
BTF-1NF	2.8 × 10^−9^	4.34 × 10^5^	5.3 × 10^6^	3.7 × 10^−9^	0.8
BFT-3NF	6.3 × 10^−9^	1.07 × 10^6^	9.7 × 10^6^	2.26 × 10^−10^	0.69
BFT-5NF	1.4 × 10^−11^	1.46 × 10^7^	2.4 × 10^8^	2.3 × 10^−11^	0.9

### 3.4. Bio-sensing platform construction and optimization

#### 3.4.1. Electrochemical characterizations

**Fig. 7:**
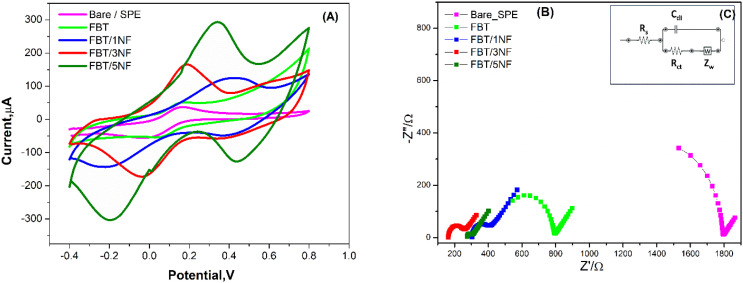
(A) CV measurements of unmodified (bare) SPE, FBT, FBT/1NF, FBT/3NF and FBT/5NF modified SPEs were conducted in a solution of standard redox probe of 5 mM ferricyanide and 0.1 M KCl as the supporting electrolyte. Scan rate of 50 mV s^−1^ was applied for all experiments. (B) EIS Nyquist spectra characterization of unmodified and modified electrodes with the nanomaterials. (C) The inset represents the modeled circuit that is used for curve fitting.

Electrochemical characterizations using CV and EIS techniques for the FBT/*x*NF nanostructures modified SPEs were studied by using ferro/ferri cyanide redox probe. Essentially, lower electrochemical signals were obtained from the unmodified SPEs, while higher signals were generated when the FBT/*x*NF nanostructures modified SPEs were used, as the increase in the oxidation/reduction peak currents was magnified. Thus, SPE surface coating with the nanocomposite enhanced the catalytic activity of the surface as shown in Fig. 7A–C, and Table 3. Surface modification of SPE with the FBT/5NF exhibited the highest redox current.

**Table 3:** Electrochemical parameters (CV & EIS) obtained from the modified screen-printed electrodes with the prepared nanomaterials. Those values are extracted from the above-discussed voltammetric as well as the impedimetric experiments (Fig. 7)

Electrode	*I*_a_ (μA)	*I*_c_ (μA)	*E*_oxd._ (V)	*E*_red._ (V)	*E*_1/2_ (V)	*R*_s_ (Ω)	*R*_ct_ (Ω)	*C* (μF)	*W* (Ω)
Bare (unmodified)	124	−158	0.274	−0.06	0.107	424	1400	0.136	0.0028
FBT	153	−188	0.233	−0.05	0.09	389	427	0.243	0.0023
FBT/1NF	159	−189	0.268	0.01	0.139	324	161	0.432	0.02216
FBT/3NF	169	−202	0.334	−0.03	0.152	186	118	0.573	0.00227
FBT/5NF	171	−216	0.227	0.045	0.136	273	88	1.231	0.0020

#### 3.4.2. Scan rate effect

**Fig. 8:**
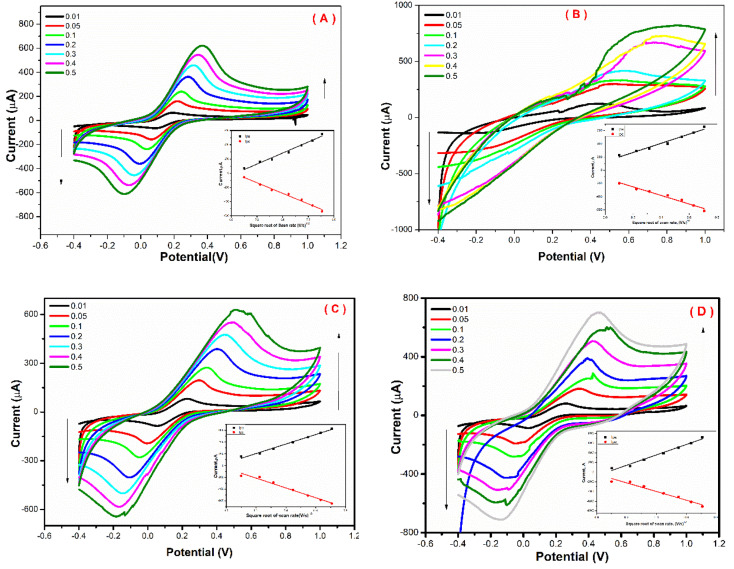
CV of prepared nanocomposites at different scan rates and influence of square root of scan rate changes on the oxidation/reduction Faradaic currents (inset figures) for (A) unmodified, (B) FBT/1NF, (C) FBT/3NF, and (D) FBT/5NF modified SPEs. Screen printed electrodes were modified with a thin film of the nanomaterials and voltammetric experiments were performed in a solution containing 5 mM ferri/ferro cyanide and 0.1 M KCl.

Electrode surfaces were coated with different Fe_0.3_BaTi_0.7_O_3_@NiFe_2_O_4_ nanocomposites (FBT, FBT/1NF, FBT/3NF and FBT/5NF) to construct an efficient enzymatic biosensor with a biocompatible interface with the glucose oxidase (GOx) redox proteins to keep the enzyme biocatalytic activity and for direct electron transfer. Thus, scan rate test was performed (from 10–500 mV s^−1^) on both electrode surfaces (modified *vs.* unmodified) as shown in Fig. 8A–D.

As a result, surface modification with the FBT/5NF provided the highest current readout activity compared with the others coating materials (*i.e.* the FBT, FBT/1NF and FBT/3NF). Consequently, the effective electrochemical active surface area (EASA) of unmodified *vs.* the modified surface with the FBT/1NF, FBT/3NF and FBT/5NF@SPE were calculated from *I*_ox_/*I*_red_*versus* square root of scan rate plot as an inset in Fig. 8A–D by using Randles–Sevcik equation ref. 40:*I*_p_ = 2.69 × 10^5^ × *n*^3/2^ × *A* × *D*^1/2^ × *Cu*^1/2^where the *I*_p_ is the peak current in amperes, *n* is the number of electron transfer, *D*: diffusion coefficient (cm^2^ s^−1^), *A*: electrochemical active area (cm^2^), *C*: concentration of the [Fe(CN)_6_]^3−/4−^ molecules (mol l^−1^), and *u:* scan rate (V s^−1^). Generated values of *I*_pa_*versus* the *u*^1/2^ can be obtained from slope value of Fig. 8. The calculated active surface area of the unmodified surface was 12.4 cm^2^, while the active surface areas of modified surfaces were 12.6 cm^2^ for FBT/1NF, 12.8 cm^2^ for FBT/3NF and 17.1 cm^2^ for modified FBT/5NF@SPE. These values indicated enlargement of modified surfaces with the nanocomposites.

#### 3.4.3. Enzymatic-based bio-sensing platform

To construct glucose biosensor with high bioactivity and high performance, the glutaraldehyde (GA 2.5%) crosslinking was implemented for GOx enzyme immobilization on the FBT/NF@SPE surface. The active enzyme (GOx) was immobilized onto the nanostructured sensor surface through the conjugation with the glutaraldehyde (GA). The biosensor preparation scheme was illustrated in Scheme 1.

For evaluating the biosensor performance, direct electron transfer was measured from the oxidation of glucose to gluconic acid and generation of peroxide as an electrochemically active bi-product to be oxidized at the active nanomaterial substrate^41–44^ as shown in eqn (1).1
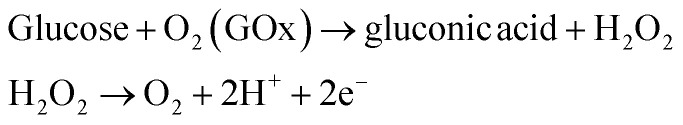


##### 3.4.3.1 Electrochemical characterization of the enzymatic biosensor platform

**Fig. 9:**
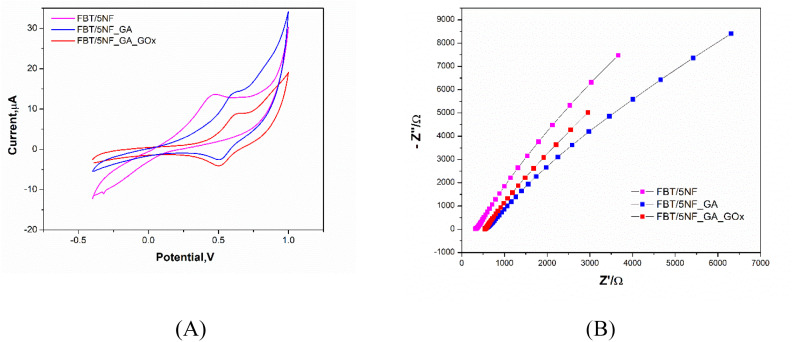
(A) CV and (B) EIS measurements of the GOx enzyme immobilization steps on the FBT/5NF@SPE surface in a solution containing standard redox probe of 5 mM ferricyanide and KCl as the supporting electrolyte. Scan rate of 50 mV s^−1^ was applied for all experiments.

The sequences of GOx enzyme immobilization on the FBT/5NF@SPE surface by glutaraldehyde (2.5%) as crosslinker were studied by cyclic voltammetry and EIS spectra as shown in Fig. 9 and Table 4. First, 5 μl of 5 mg FBT/5NF suspended in 1 ml double distilled water was drop casted on the SPE surface then immobilize 5 μl of GOx enzyme (500 μg/1 ml PBS buffer) on FBT/5NF@SPE surface by glutaraldehyde (2.5%) as crosslinker and left to dry at room temperature. The layer-by-layer sensor preparation was characterized by FCN redox probe solution using cyclic voltammetry (CV) and electrochemical impedance spectroscopy (EIS) techniques. For cyclic voltammetry studies (see Fig. 9A), every layer drop-casted on the SPE surface led to a decrease in the redox peak current of FCN solution due to the blocking of active surface area with the enzyme layer. This decrease in the voltammetric signals, combined with an increase in the impedimetric responses (see Fig. 9B and Table 4) which indicated the chemical immobilization and stability of the enzymatic layer onto the nanostructured surface.

**Table 4:** The electrochemical parameters (CV & EIS) obtained for the modified electrodes with the GOx@GA/FBT/5NF@SPE. Those values are extracted from the above-discussed voltammetric as well as the impedimetric experiments (Fig. 9)

Electrode type	*I*_a_ (μA)	*I*_c_ (μA)	*E*_oxd._ (V)	*E*_red._ (V)	*E*_1/2_ (V)	*R*_s_ (Ω)	*R*_ct (1)_ (Ω)	CPE (μF)	*n*	*W* (Ω)
FBT/5NF	14.02	1.5	0.45	0.46	0.45	328.05	290.06	4.99	0.74	11.08
FBT/5NF@GA	14.8	−2.9	0.58	0.52	0.55	560.7	44 053	8.06	0.77	4.62
FBT/5NF@GA@GOx	8.75	−4.1	0.61	0.51	0.56	547.7	81 864	11.3	0.78	0.432

##### 3.4.3.2 Influence of FBT/NF nanocomposite ratio on the enzymatic biosensor sensitivity

**Fig. 10:**
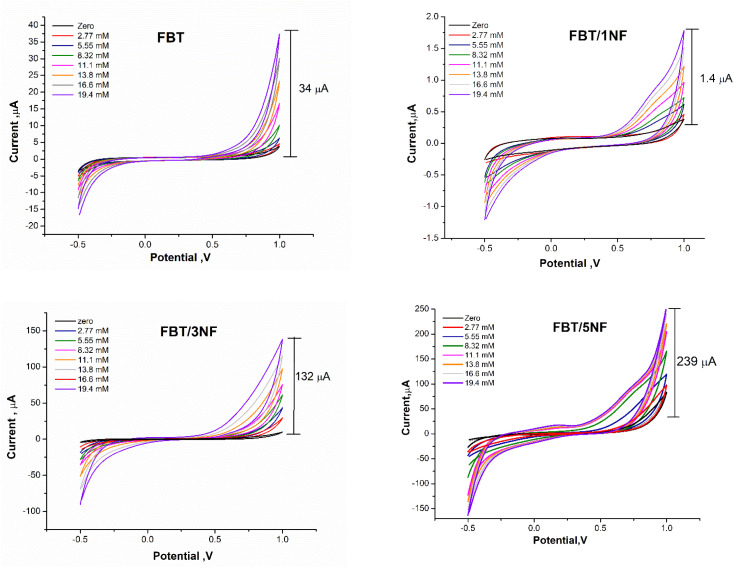
CVs response of GOx@GA@FBT/NF@SPEs biosensor's containing different ratios of FBT/*x*NF nanocomposites (FBT; FBT:1NF; FBT:3NF; or FBT:5NF) and fixed concentration of GOx with500 μg ml^−1^ and 2.5% GA. The voltametric scans was carried out by using PBS buffer (pH = 7.4) solution containing different concentrations of glucose from 2.77–19.4 mM.

The enzymatic bio-sensing performance of different ratios of nanocomposite modified SPE surfaces was studied whereas the 5 μl of GOx enzyme (500 μg ml^−1^) was immobilized after drop cast 5 μl GA (2.5%) on sensor chips surface which modified with 5 μl of 25 mg ml^−1^ FBT, FBT/1NF, FBT/3NF, or FBT/5NF. As a result, cyclic voltammetry graphs showed an obvious response towards the addition of glucose concentration (the targeting analyte) with all modified surfaces, while the highest voltammetric oxidation peak current feedback was presented by the GOx@GA@FBT/5NF@SPE surface (see Fig. 10). Accordingly, the GOx immobilization by GA onto the FBT/5NF-based SPE surface was assigned for the glucose biosensing platform construction.

**Fig. 11:**
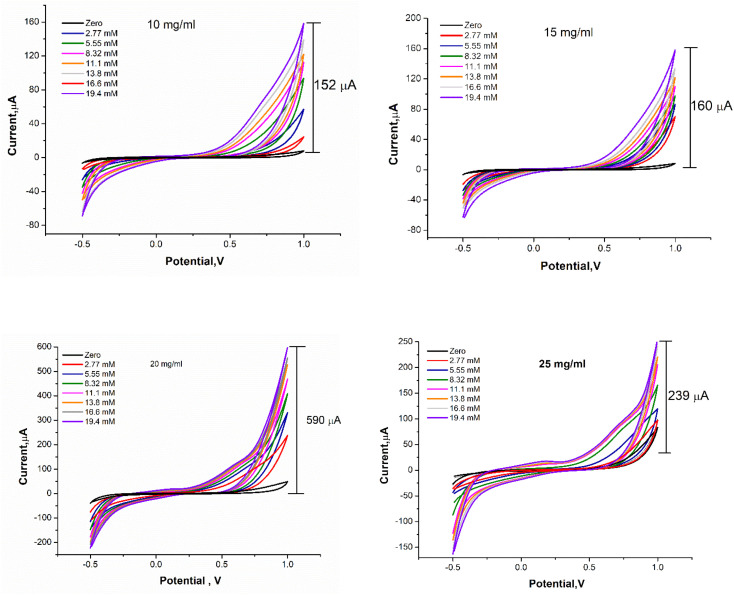
CVs response of GOx@GA@FBT/5NF@SPEs biosensor's containing different concentrations (10,15,20 and 25 mg ml^−1^) of FBT/5NF nanocomposite and fixed concentration of GOx with500 μg ml^−1^ and 2.5% GA. The voltammetric scans was carried out by using PBS buffer (pH = 7.4) solution containing different concentrations of glucose from 2.77–19.4 mM.

Further optimization was carried out whereas the concentration of FBT/5NF nanocomposite was studied over a range of concentration (10, 15, 20 and 25 mg ml^−1^) in GOx@GA@FBT/5NF@SPE surface. As shown in Fig. 11, the concentration of 20 mg ml^−1^ of FBT/5NF presented the highest oxidation peak current readout against different additions of glucose.

### 3.5. Chronoamperometric measurements

To optimize the fabricated biosensor response toward glucose detection, different parameters were studied including accumulation potential, enzyme concentration and pH effect of supporting electrolyte.

#### 3.5.1. Effects of accumulation potential

**Fig. 12:**
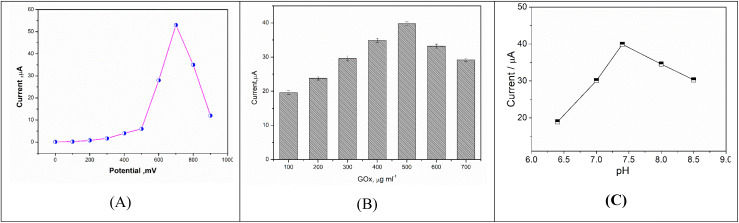
(A) Effect of accumulation potential on the chronoamperometric current responses of the GOx-based biosensor in a solution of PBS (pH = 7.4) containing 0.83 mM glucose. (B) Effect of GOx concentration on the biosensor current response by using PBS buffer (pH = 7.4) containing 0.69 mM of glucose. (C) Effect of different pHs of phosphate buffer on the glucose biosensors current response in the presence of glucose with a concentration of 0.69 mM.

To choose a single defined applied DC working potential, GOx-based biosensor was tested, while the chronoamperometric measurements were conducted at different applied potential values (from 0.0 mV to 1000 mV). As shown in Fig. 12A, the increase in the oxidation current response was dependent on the applied voltage, however the highest pronounced increase was achieved at 700 mV. Exceed this value of applied voltage led to a decrease in the oxidation current. Thus, 700 mV was selected as the working potential.

#### 3.5.2. Influence of GOx concentration

Here, the influence of the immobilized GOx concentration onto the sensor surface was tested over a wide range (from 100 to 700 μg ml^−1^) to define the best concentration that provides high response. As a result, the current response values were dependent on the concentration of GOx enzyme as shown in Fig. 12B. It is clear from the graph that the current value of GOx biosensor increased in respect to the increase in the GOx loading until reaching 500 μg ml^−1^. Exceeding this concentration led to a disturbance in the electrochemical signals due to the enzyme steric hindrance. Therefore, the best immobilized GOx enzyme concentration provides high response is 500 μg ml^−1^ which selected for further experimental work.

#### 3.5.3. Influence of pH

The effect of buffer solution pHs on the biosensor response was studied over a range between pH 6.4–8.5 at a fixed applied potential 0.7 V. As it is shown in Fig. 12C, the current response increased as the pH value increased from 6.4–7.4 then decreased at pH 8.0. Therefore, the highest amperometric response was obtained at pH 7.4.

#### 3.5.4. Calibration curve

**Fig. 13:**
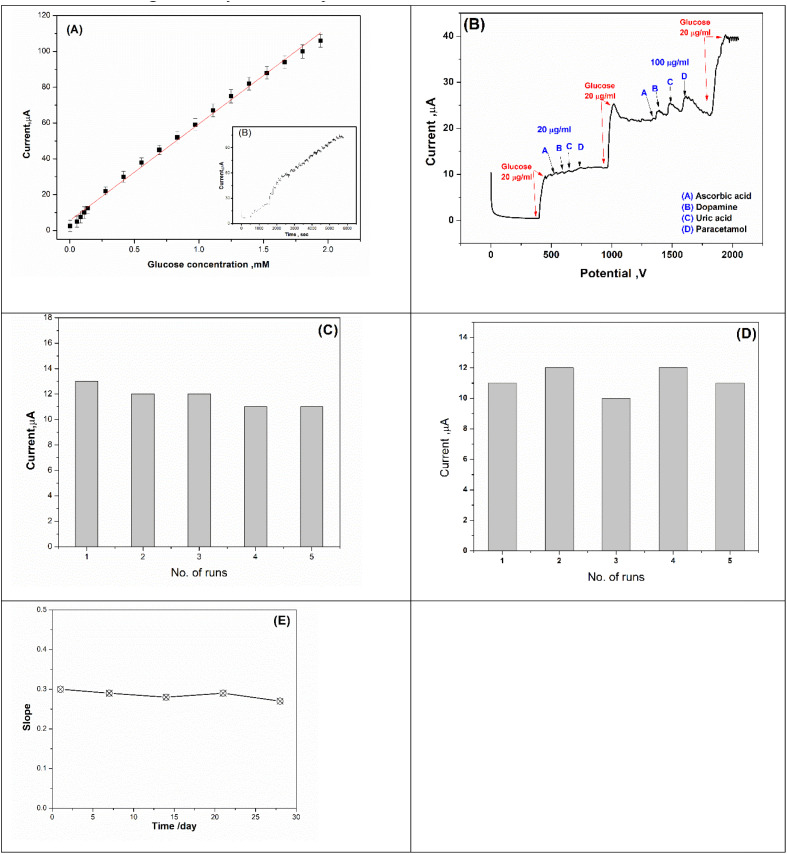
(A) Calibration curve of GOx@GA@FBT/5NF@SPE biosensor for chronoamperometric glucose detection in PBS buffer pH = 7.4 at fixed potential of 0.7 V (the chronoamperometric measurement presented as inset figure). (B) Selectivity study of the proposed biosensor (GOx@GA@FBT/5NF@SPE) response towards glucose and non-targeting analytes (such as dopamine, ascorbic acid, paracetamol and uric acid). (C) Reproducibility, (D) repeatability and (E) life time of GOx@GA@FBT/5NF@SPE biosensor using a solution of PBS buffer (pH = 7.4) containing 20 μM glucose and chronoamperometric detection at fixed potential 0.7 V.

To define the sensitivity of this biosensing assay and its limit of detection, the newly prepared GOx-biosensor chips (GOx@GA@FBT/5NF@SPE) were tested over a wide range of glucose concentration. Hence, chronoamperometric signals (inset Fig. 13A) were collected, and the oxidation current generated from each measurement were plotted in Fig. 13A showing a linear relationship with the concentration range of 0.0027–1.9 mM, and regression coefficient of *R*^2^ value 0.9968. From this dynamic calibration curve, a calculated detection limit (LOD) of 0.5 μM was obtained. In addition to this high sensitivity (757.14 μA mM^−1^ cm^−2^), the fast responses obtained after every 50 second from each addition of glucose are reflecting the synergetic catalytic activity acquired by the utilization of the nanocomposite as the main sensing approach. Referring to the previously reported electrochemical bio-sensing methods for glucose detection, a survey was tabulated (Table 5), whereas the current assay is exhibiting high sensitivity among many other methods.

**Table 5:** Comparison of analytical parameters for detection of glucose over various modified electrodes^a^

Electrode composite	Applied potential	Linearity (mM)	LOD (μM)	Sample	Life time	Ref.
Nafion/GOx/carbon fibre-hemain AuNP/graphite electrode	−0.10 V (*vs.* Ag/AgCl)	0.1–0.9	5 0	—	—	45
Pencil graphite electrode modified with naphthalenedimide/3,4-ethylenedioxythiophene conjugated polymer and enrichedwith Au nanoparticles	−0.7 V (Ag/AgCl)	0.009–0.33	40	Orange juice	3 weeks	46
Au@rGO/PIn/ferritin/GOx/GCE	—		—	—	—	47
GOx-chitosan/rGOAuNPs/	−0.30 V(*vs.* Ag/AgCl)	0.1–1.3	76	—	36 days	48
Graphite NPs-pyrene-GOx/GCE	0.60 V (*vs.* Ag/AgCl)	0–2.2 7	50	Urine	30 days	49
GOx-graphene-thiol/Au nanocube/Au disk	−0.40 V (*vs.* Ag/AgCl)	0–0.8	—	—	14 days	50
GOD-CS/AgNWs/GCE	−0.15 V (*vs.* saturated calomel electrode)	0.01–0.8	2.83	Human blood glucose	10 days	51
Nafion/GOx/Au–Ni coaxial nanorod array/Au electrode	0.40 V (−)	0.028–27.5	5.5	—	30 days	52
GOx-Au/Pd-MWCNT/SPCE	—	—	70	Human blood glucose	14 days	53
GOx@GA@FBT/5NF@SPE	0.7 V (*vs.* Ag)	0.0027–1.9	0.50	Human blood glucose	30 days	This work

a Abbreviations mentioned in the table: PENDI: polymerized *N*,*N*_0_-bis(2-hexyl)-2,6-(3,4 ethylene dioxy-thiophene)-1,4,5,8-naphthalenimide; PGE: pencil graphite electrode, rGO: reduced graphene oxide, Pin: polymerization of indole, CS: chitosan, PEI: poly-ethylenimine.

### 3.6. Selectivity testing

For selectivity study, the proposed biosensor (GOx@GA@FBT/5NF@SPE) response towards non-targeting analytes (such as dopamine, ascorbic acid, paracetamol and uric acid) were tested, as shown in Fig. 13B. The enzymatic chronoamperometric feedback was only positive toward the glucose addition, while exposing the biosensor chips toward the other foreign molecules did exhibit reasonable electrochemical signals.

### 3.7. Repeatability, reproducibility and lifetime

The repeatability of the prepared biosensor (GOx@GA@FBT/5NF@SPE) was studied by measuring the amperometric response toward 20 μM glucose using the same electrode for six consecutive measurements (see Fig. 13C). The results showed a tiny variation (1.6%) which indicates the high repeatability of the sensing chips.

Furthermore, the reproducibility testing was carried out using five different freshly prepared sensor chips, whereas each chip was exposed to glucose concentration (20 μM) while the electrochemical signals were collected from all chips (Fig. 13D). As a result, a small variation among all tested chips (2.3%) was obtained. Eventually, the lifetime of the prepared and stored biosensor chips was tested, whereas a batch (a group of) chips was prepared and stored at 4 °C, while the electrochemical performance of each chip was tested over different duration (as shown in Fig. 13E). The results showed a high stability for the chips.

### 3.8. Application for glucose detection in blood samples

In reality, glucose monitoring for health care is very crucial. Therefore, the newly developed sensor chips were applied for rapid detection of glucose in blood samples. As shown in Table 6, high recovery was obtained (90–110%); whereas all analyzed samples were confirmed with a reference laboratory test.

**Table 6:** Determination of glucose concentration in different blood samples using the currently developed biosensors and a reference laboratory method for the validation and confirmation

Sample no. (Age/year)	Biosensor mg dl^−1^ ± SD^a^	Laboratory mg dl^−1^	Recovery%
1 (16)	110 ± 4.0	100	110
2 (30)	135 ± 6.0	140	96
3 (25)	95 ± 6.0	100	95
4 (42)	165 ± 8.0	153	107
5 (33)	134 ± 5.0	130	103
6 (65)	170 ± 3.0	167	101
7 (45)	195 ± 7.0	203	96
8 (21)	92 ± 6.0	102	90
9 (41)	105 ± 9.0	115	91
10 (53)	210 ± 5.0	214	98

a SD: standard deviation.

## 4 Conclusion

Multiferroic nano-hexagonal FBT@NF composites were fabricated *via* solgel chemical reactions of sol nickel ferrite as coated layer and iron barium titanate@nickel ferrite powders. XRD analysis validates the successful fabrication of the di-phase FBT and NF composite nanomaterial with high crystalline degree. The TEM supports the well-organized hexagonal structure of FBT/NF, with highly ordered hexagonal lattice channels, providing clear visualization of the structural precision within the material.

The conductivity of the ferroelectric/paraelectric (FBT/NF) composite was affected by the ratio, where the paraelectric particle hindered the interaction between the permanent dipoles of the ferroelectric. The conductivity and the Curie temperature of the composite systems decreased compared to the ferroelectric phase FBT. The electrical properties of the ferroelectric/paraelectric composites demonstrate the successful influence of each other, as well as the possibility of benefiting from the combination of anisotropic materials to produce materials with new properties that combine the properties of their components.

Nanostructures-based electrochemical biosensors provide powerful platforms for constructing reliable and portable biosensing devices. Thus, in the current study, the use of SPE for entrapping of a catalytically active enzyme (GOx) on the new nanostructures FBT/NF was performed. The developed sensor can be used for glucose detection with a wide linear of 2.7–1900 μM with a detection limit of 0.5 μM by using chronoamperometric technique. The biosensor was applied for glucose detection in blood samples without any treatment before analysis with high recovery compared with reference method. The obtained fast and high efficiency direct electron transfer from the redox active center of immobilized enzyme and screen-printed electrode by using the new synthesized FBT/NF nanostructures is a promising avenue for fabricating further excellent electrochemical biosensors. Which could be useful for monitoring cancer and infectious disease, drugs and analyzing disease biomarkers.

## Data availability

The authors confirm that the data supporting the findings of this study are available within the article.

## Conflicts of interest

The authors declare that they have no known competing financial interests or personal relationships that could have appeared to influence the work reported in this paper.
